# Sex differences in extracorporeal cardiopulmonary resuscitation for out-of-hospital cardiac arrest: nationwide multicenter retrospective study in Japan

**DOI:** 10.1186/s13054-024-05086-9

**Published:** 2024-10-31

**Authors:** Akira Kawauchi, Yohei Okada, Makoto Aoki, Akihiko Inoue, Toru Hifumi, Tetsuya Sakamoto, Yasuhiro Kuroda, Mitsunobu Nakamura, the SAVE-J II Study Group

**Affiliations:** 1 Department of Critical Care and Emergency Medicine, Japanese Red Cross Maebashi Hospital, Maebashi, Gunma Japan; 2 Health Services and Systems Research, Duke-NUS Medical School, Singapore, Singapore; 3 Division of Traumatology, Research Institute, National Defense Medical College, Saitama, Japan; 4 Department of Emergency and Critical Care Medicine, Hyogo Emergency Medical Center, Kobe, Japan; 5 Department of Emergency and Critical Care Medicine, St. Luke’s International Hospital, Tokyo, Japan; 6 Department of Emergency Medicine, Teikyo University School of Medicine, Tokyo, Japan; 7 Department of Emergency, Disaster and Critical Care Medicine, Kagawa University Hospital, Kagawa, Japan

**Keywords:** Extracorporeal cardiopulmonary resuscitation, Out-of-hospital cardiac arrest, Sex differences

## Abstract

**Background:**

Previous studies examining sex differences in patients undergoing extracorporeal cardiopulmonary resuscitation (ECPR) for out-of-hospital cardiac arrest (OHCA) have indicated that women have favorable outcomes; however, detailed evidence remains lacking. We aimed to investigate sex differences in the backgrounds and outcomes of patients undergoing ECPR for OHCA.

**Methods:**

This study was a secondary analysis of the registry from the SAVE-J II study, a retrospective multicenter study conducted in Japan from 2013 to 2018. Adult patients without external causes who underwent ECPR for OHCA were included. The primary outcome was a favorable neurological outcome (Cerebral Performance Status 1 or 2) at hospital discharge. We used multilevel logistic regression to evaluate the association of sex differences, adjusting for center-level (hospital) and individual-level variables (patient background, cardiac arrest situation, and in-hospital intervention factors). For sensitivity analyses, we performed three models of multilevel logistic regression when selecting confounders.

**Results:**

Among the 1819 patients, 1523 (83.7%) were men, and 296 (16.3%) were women. The median age (61.0 vs. 58.0 years), presence of a witness (78.8% vs. 79.2%), and occurrence of bystander CPR (57.5% vs. 61.6%) were similar between groups. Women were more likely to present with an initial non-shockable rhythm (31.7% vs. 49.7%), as well as a non-shockable rhythm at hospital arrival (52.1% vs. 61.5%) and at ECMO initiation (48.1% vs. 57.1%). The proportion of favorable neurological outcomes was 12.3% in males and 15.9% in females (*p* = 0.10). Multilevel logistic regression analysis showed that the female sex was significantly associated with a favorable neurologic outcome at discharge (adjusted odds ratio: 1.60 [95% confidence interval: 1.05–2.43]; *p* = 0.03). This advantage in women was consistently observed in the sensitivity analyses.

**Conclusions:**

The female sex is significantly associated with favorable neurological outcomes at hospital discharge in patients who received ECPR for OHCA.

**Supplementary Information:**

The online version contains supplementary material available at 10.1186/s13054-024-05086-9.

## Introduction

Sex differences and gender gaps are of increasing concern and interest in critical care research [1, 2]. Research on sex differences in biological factors (e.g., sex hormones) and gender gaps in sociocultural factors (e.g., treatment gaps) is crucial even in out-of-hospital cardiac arrest (OHCA) studies [3]. Generally, women present with poorer prognostic outcomes than men in the overall OHCA population [4]. However, a recent systematic review revealed no significant sex-related differences in neurological outcomes after adjusting for potential confounders [5]. This suggests that outcome differences may be influenced more by background factors and treatment gaps than by inherent biological sex differences.

ECPR is an emerging life-saving technique for patients with refractory cardiac arrest [6, 7], and its use has been increasing over time [8]. A recent systematic review of seven observational studies demonstrated that the female sex is associated with favorable outcomes in patients receiving ECPR for OHCA, suggesting the possibility of a prognostic advantage for women [9]. However, each included odds ratio (OR) was not sufficiently reliable for estimating the association. This is because the estimated OR for sex was calculated as one of the covariates in various models for confounding adjustments from different studies. For robust evidence of the advantages for women, a focused study on sex differences in patients undergoing ECPR for OHCA is warranted. Although several previous studies investigated sex differences in patients requiring extracorporeal membrane oxygenation (ECMO) support, these studies included patients other than OHCA. Therefore, despite the implication of women’s advantage in neurological outcomes, studies investigating the sex differences in outcomes, specifically in patients undergoing ECPR for OHCA, remain lacking.

This study aimed to reveal sex differences in the background, treatment gaps, and outcomes of patients with OHCA who underwent ECPR and to investigate the effect of biological sex differences on outcomes by adjusting for confounders using a large and detailed registry of ECPR for OHCA with rigorous methodology.

## Methods

### Study design and data collection

This study was a secondary analysis of a multicenter, retrospective, observational registry from the SAVE-J II study. The SAVE-J II study collected data on adult patients (≥ 18 years old) who received ECPR owing to OHCA in 36 intensive care units (ICUs) in Japan from 2013 to 2018 [10]. ECPR was defined as the resuscitation of patients with OHCA with veno-arterial extracorporeal membrane oxygenation. Pre-hospital and in-hospital data were recorded by the treating physicians at each participating institution. The study protocol was approved by the ethical review board of the Maebashi Red Cross Hospital (approval number: 2023–52), and the requirement for written informed consent was waived. This study followed the reporting recommendations on strengthening the reporting of observational studies in epidemiology (STROBE) statement (Table S1) [11].

### Patient selection and outcomes

Patients in the SAVE-J II study underwent eligibility screening excluding those with external causes of OHCA (e.g., accidental hypothermia, drug, heatstroke, trauma, drowning, or suffocation) and those with ROSC at hospital arrival and ROSC at ECMO initiation. Patients who transferred from another hospital and patients with unknown outcomes were also excluded. ECPR implementation varied at the discretion of the clinician or facility protocol, lacking standardized criteria for ECPR or extracorporeal membrane oxygenation management among the study groups. The primary outcome was a favorable neurological outcome at discharge, defined as Cerebral Performance Category 1 or 2 [12]. Secondary outcomes included hospital survival, withholding/withdrawal of life-sustaining therapy (WLST), and severe acute kidney injury (AKI) during ECMO. WLST was defined as the decision to withhold or withdraw medical interventions (including ECMO or other treatments) for patients expected to have poor outcomes despite further treatment. In Japan, this typically integrates patient advance directives with input from multidisciplinary staff and family members. Reasons for WLST included perceived unfavorable prognosis, inability to sustain ECMO support, management of complications, and other factors [13]. AKI was defined according to the Kidney Disease Improving Global Outcomes (KDIGO) guidelines [14]. Severe AKI, defined as stage 3 AKI in KDIGO criteria required a 3.0-fold increase in creatinine or serum creatinine ≥ 4.0 mg/dL initiation of renal replacement therapy, urine output < 0.3 mL/kg/h for > 24 h, or anuria for > 12 h [14]. ECMO duration, ICU stay duration, and hospital stay duration were also collected.

### Measurement

From SAVE-J II study registry, we collected several variables as confounding factors: age, sex, body mass index (BMI), past medical history (hypertension, diabetes mellitus, cardiac disease, stroke, chronic kidney disease), performance status [15], location and cause of cardiac arrest, presence of witness and bystander CPR, use of automated external defibrillator (AED), initial cardiac rhythm, cardiac rhythm at hospital survival and ECMO initiation, time from emergency call to hospital arrival, low-flow time (time from cardiac arrest to ECMO initiation), TiPS65 score [16], clinical characteristics at hospital arrival (signs of life, body temperature, pH, and lactate), coronary angiography (CAG), percutaneous coronary intervention (PCI), intra-aortic balloon pumping (IABP), and targeted temperature on ECMO. The TiPS65 score is a validated scoring scale used to predict outcomes in patients with OHCA with an initial shockable rhythm based on four variables (time to hospital < 25 min, pH value in blood gas assessment > 7.0, shockable upon hospital arrival, and < 65 years old) [16, 17]. Signs of life were defined as the presence of gasping, pupillary light reaction, or Glasgow Coma Scale-Motor Score ≥ 2 or higher [18].

### Statistical analyses

Patients were divided into male and female groups, and their characteristics and outcomes were presented descriptively. Categorical variables were presented as numbers and percentages, and continuous variables were presented as medians and interquartile ranges. Significant differences were reported as *p* < 0.05. To address missing data bias, we performed multiple imputations using a Markov chain Monte Carlo method known as chained equation imputation, generating 20 completed datasets [19]. We did not estimate a sample size before analysis because this study was a secondary analysis of an available database [11].

Multilevel logistic regression analysis examined sex differences in outcomes using odds ratios (ORs) and 95% confidence intervals (CIs). The primary multilevel logistic regression analysis was adjusted for center-level variables (hospital) and individual-level variables (age, BMI, past medical history, performance status, location of cardiac arrest, cause of cardiac arrest, presence of witness and bystander CPR, AED, initial cardiac rhythm, low-flow time, signs of life, pH at hospital arrival, PCI, IABP, and target temperature). These variables were selected based on previous studies identifying prognostic factors and sex-related treatment disparities in OHCA settings and ECPR populations [4, 9, 20, 21]. We performed a subgroup analysis based on the cause of cardiac arrest and risk factors for ECPR in OHCA [6, 9], including age (cut-off of 70 years) [6], initial rhythm (shockable or non-shockable), bystander CPR, and low flow time (cut-off of 60 min) [6]. For sensitivity analysis regarding confounder selection, we used three models of multilevel logistic regression. These models were adjusted for various factors sets of factors: a pre-cardiac arrest factor model (age, BMI, past medical history, performance status, and cause of cardiac arrest), a before-ECPR factor model (age, BMI, past medical history, performance status, cause of cardiac arrest, location, presence of witness and bystander CPR, AED, initial cardiac rhythm, low-flow time, ROSC before cannulation, signs of life, and pH), and an after-ECPR factor model (PCI, IABP, and target temperature). In addition, to investigate the impact of ROSC on sex differences in the primary outcome, we performed another sensitivity analysis including patients with ROSC at hospital arrival and at ECMO initiation, who were previously excluded according to the exclusion criteria in this study. Finally, we performed an additional analysis to explore the underlying mechanism of sex differences in primary outcomes, incorporating severe AKI alongside other variables in the main analysis. The decision was informed by a previous study on ECPR which demonstrates a higher incidence of AKI in men [22], a factor associated with poor outcomes following cardiac arrest [23–25]. All statistical analyses were conducted using Stata software (version 17.0; Stata Corp, College Station, TX, USA).

## Results

### Patient characteristics and outcomes

**Fig. 1:**
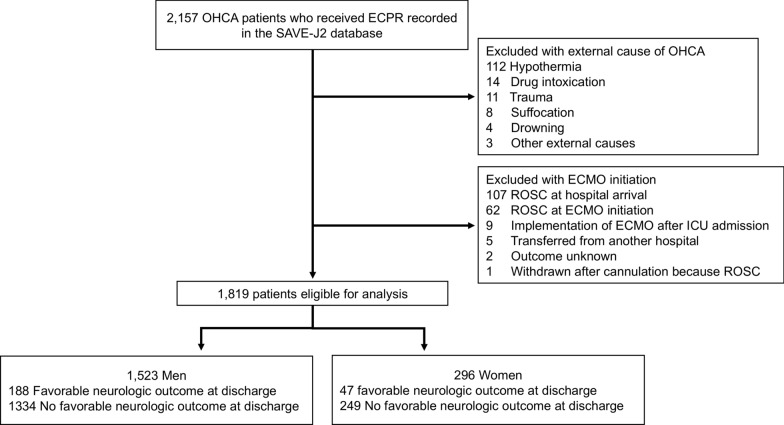
Patient flow chart. OHCA, out-of-hospital cardiac arrest; ECPR, extracorporeal membrane oxygenation; ECMO, extracorporeal membrane oxygenation

Among the 2157 patients recorded in the SAVE-J II registry, 1819 were eligible for this study (Fig. 1). A total of 338 patients were excluded. Of the eligible patients, 1523 were men, and 296 were women (Tables 1 and 2 and, Tables S2 and S3). Patient characteristics and outcomes are presented in Tables 1 and 2, respectively. The median age was identical between the two groups (61.0 [50.0–68.0] vs. 58.0 [46.0–69.0] years old, *p* = 0.24). The male group had a higher BMI (24.6 [22.0–27.2] vs. 23.5 [20.3–27.1], *p* = 0.01), more past medical histories, and better performance status. Although cardiac arrests most frequently occurred at home (37.8% vs. 51.4%, *p* < 0.001) in both groups, they were more common in public places, streets, and workplaces among men. The most common cause of cardiac arrest in both groups was acute coronary syndrome (57.1% vs. 34.1%, *p* < 0.001). The proportions of witnessed cardiac arrests and bystander CPR were identical in both groups. Men more frequently presented with a shockable rhythm at the initial rhythm, upon hospital arrival, and ECMO initiation. Low-flow time was similar (55.0 [45.0–66.0] vs. 55.0 [46.0–65.5] min, *p* = 0.91) between the two groups. The TiPS65 score, indicating the degree of favorable prognostic factors, was higher in the male group (2.0 [1.0–2.0] vs. 1.0 [1.0–2.0], *p* < 0.001). Men underwent CAG, PCI, and IABP more frequently.

**Table 1:** Patient characteristics before hospital arrival

	Men (n = 1523)	Women (n = 296)	*p*-value
Age (year)	61.0 [50.0–68.0]	58.0 [46.0–69.0]	0.24
Body mass index	24.6 [22.0–27.2]	23.5 [20.3–27.1]	0.01
Past medical history			
Hypertension	474 (31.1%)	85 (28.7%)	0.41
Diabetes mellitus	309 (20.3%)	39 (13.2%)	0.004
Cardiac disease	374 (24.6%)	64 (21.6%)	0.28
Stroke	93 ( 6.1%)	19 ( 6.4%)	0.84
Chronic kidney disease	80 ( 5.3%)	10 ( 3.4%)	0.17
Performance Status*			0.13
0	1,339 (87.9%)	248 (83.8%)	
1	109 ( 7.2%)	31 (10.5%)	
2	25 ( 1.6%)	8 ( 2.7%)	
Location of cardiac arrest			< 0.001
Home	575 (37.8%)	152 (51.4%)	
Public place/Street	501 (32.9%)	60 (20.3%)	
Workplace	186 (12.2%)	15 ( 5.1%)	
Ambulance**	158 (10.4%)	52 (17.6%)	
Other	97 ( 6.4%)	17 ( 5.7%)	
Cause of cardiac arrest			< 0.001
Acute coronary syndrome	869 (57.1%)	101 (34.1%)	
Arrhythmia	191 (12.5%)	42 (14.2%)	
Myocarditis/Myopathy	92 ( 6.0%)	23 ( 7.8%)	
Aortic dissection	94 ( 6.2%)	18 ( 6.1%)	
Pulmonary embolism	29 ( 1.9%)	30 (10.1%)	
Other diagnosed internal disease	136 ( 8.9%)	63 (21.3%)	
Unknown etiology	111 ( 7.3%)	19 ( 6.4%)	
Witness	1,197 (78.8%)	232 (79.2%)	0.87
Bystander CPR	863 (57.5%)	178 (61.6%)	0.19
Automated external defibrillator	968 (64.0%)	147 (50.7%)	< 0.001
Initial cardiac rhythm			< 0.001
Shockable rhythm	1,028 (67.5%)	144 (48.6%)	
PEA	359 (23.6%)	115 (38.9%)	
Asystole	124 ( 8.1%)	32 (10.8%)	

Data are presented as median [interquartile range] or n (%)

CPR, cardiac pulmonary resuscitation; ROSC, return of self-circulation; PEA, pulseless electrical activity

^*^ Eastern Cooperative Group Performance Status

^**^ Cardiac arrest occurred during transport from emergency medical staff arrival to hospital arrival

**Table 2:** Patient characteristics after hospital arrival and outcomes

	Men (n = 1,523)	Women (n = 296)	*p*-value
Cardiac rhythm at hospital arrival			0.012
Shockable rhythm	725 (47.6%)	113 (38.2%)	
PEA	485 (31.8%)	121 (40.9%)	
Asystole	309 (20.3%)	61 (20.6%)	
Cardiac rhythm at ECMO initiation			
Shockable rhythm	778 (51.1%)	119 (40.2%)	< 0.001
PEA	492 (32.3%)	126 (42.6%)	
Asystole	241 (15.8%)	43 (14.5%)	
Time from emergency call to hospital arrival (min)	33.0 [26.0–42.0]	35.0 [26.0–44.0]	0.44
Low-flow time* (min)	55.0 [45.0–66.0]	55.0 [46.0–65.5]	0.91
Tips65 score**	2.0 [1.0–2.0]	1.0 [1.0–2.0]	< 0.001
Clinical characteristics at hospital arrival			
Signs of life***	177 (18.8%)	35 (20.5%)	0.6
Gasping	142 (10.6%)	28 (10.6%)	0.97
Pupillary light reaction	90 ( 8.3%)	20 (10.3%)	0.37
GCS M > 1	21 ( 1.4%)	3 ( 1.0%)	0.61
Body temperature (℃)	35.2 [34.2–35.9]	35.3 [34.5–35.9]	0.3
pH	6.9 [6.8–7.0]	6.9 [6.8–7.0]	0.19
Lactate (mmol/L)	13.0 [10.1–16.0]	12.8 [10.1–15.0]	0.15
Coronary angiography	1,146 (75.3%)	200 (67.6%)	0.006
Percutaneous coronary intervention	691 (46.9%)	75 (26.0%)	< 0.001
Intra aortic balloon pumping	936 (61.7%)	147 (49.7%)	< 0.001
Target temperature (℃)			
≦34	662 (43.5%)	119 (40.2%)	0.4
35	91 ( 6.0%)	21 ( 7.1%)	
36≦	257 (16.9%)	44 (14.9%)	
Outcomes			
Favorable neurologic outcome at discharge	188 (12.3%)	47 (15.9%)	0.10
Hospital survival	373 (24.5%)	83 (28.0%)	0.20
Severe acute kidney injury	240 (20.2%)	29 (12.9%)	0.01
Withhold/withdraw life-sustaining therapy	413 (27.1%)	88 (29.7%)	0.36
Perceived unfavorable prognosis	330 (21.7%)	76 (25.7%)	0.37
Inability to sustain ECMO run or manage complications	72 ( 4.7%)	11 ( 3.7%)	
Other reason	11 ( 0.7%)	1 ( 0.3%)	
ECMO duration	3.0 [2.0–5.0]	3.5 [2.0–5.0]	0.46
ICU duration	3.0 (1.0–9.0)	3.0 (1.0–10.0)	0.63
Hospital duration	3.0 (1.0–17.0)	3.0 (1.0–19.0)	0.22

PEA, pulseless electrical activity; ECMO, extracorporeal membrane oxygenation; GCS, Glasgow Coma Scale-Motor; ICU, intensive care unit

^*^Time from cardiac arrest or collapse to ECMO run

^**^Tips65 includes four variables (time to hospital shorter than 25 min, pH value in blood gas assessment higher than 7.0, shockable on hospital arrival, and younger than 65 years old)

^***^Signs of life were defined as a composite of any of the following: gasping, pupillary light reaction, or a Glasgow Coma Scale-Motor Score of greater than or equal to 2

### Primary outcome

**Fig. 2:**
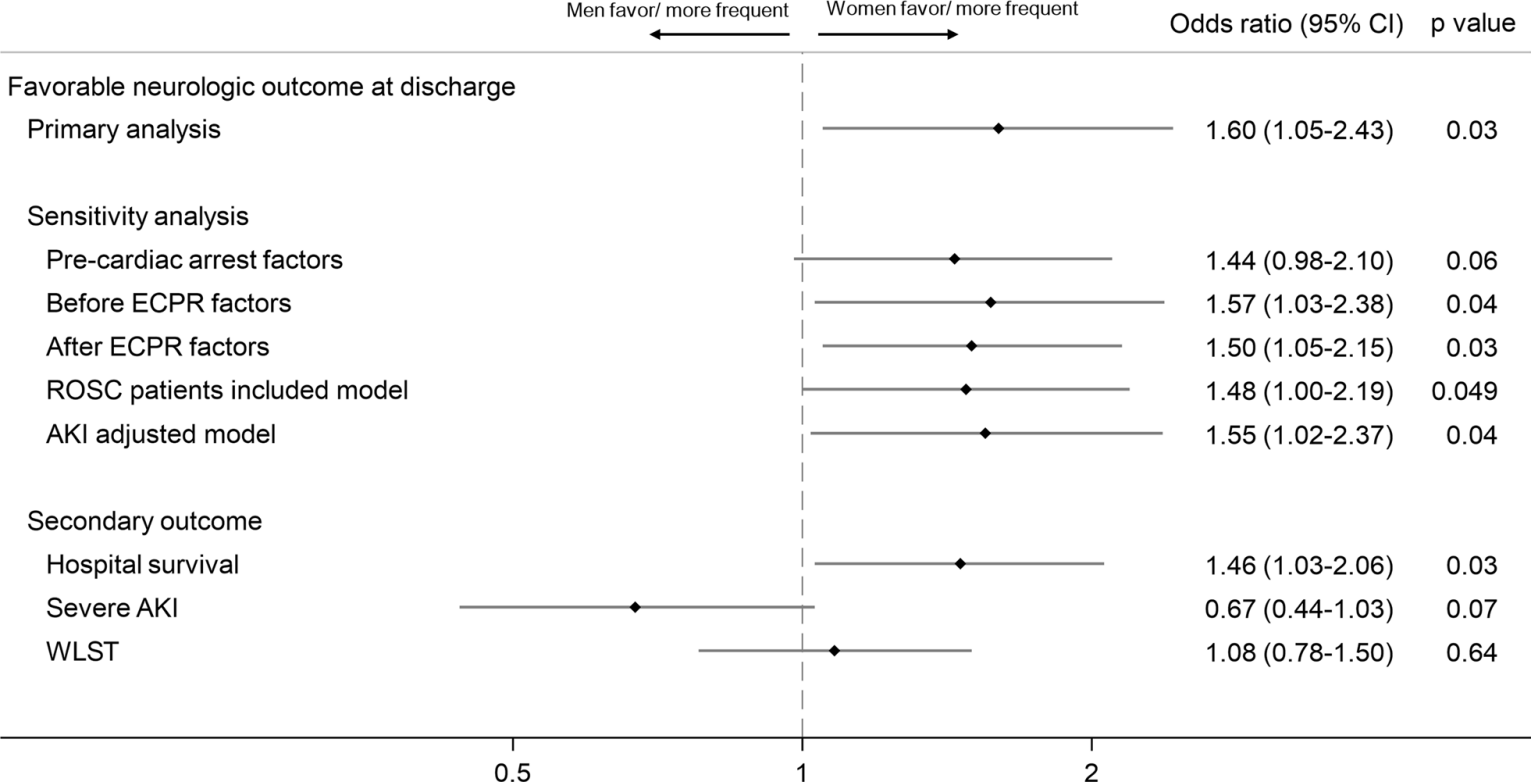
Results of multilevel logistic regression analysis. ECPR, extracorporeal membrane oxygenation; ROSC, return of spontaneous circulation; AKI, acute kidney injury; WLST, withhold/withdraw life-sustaining therapy; CI, confidential interval

**Fig. 3:**
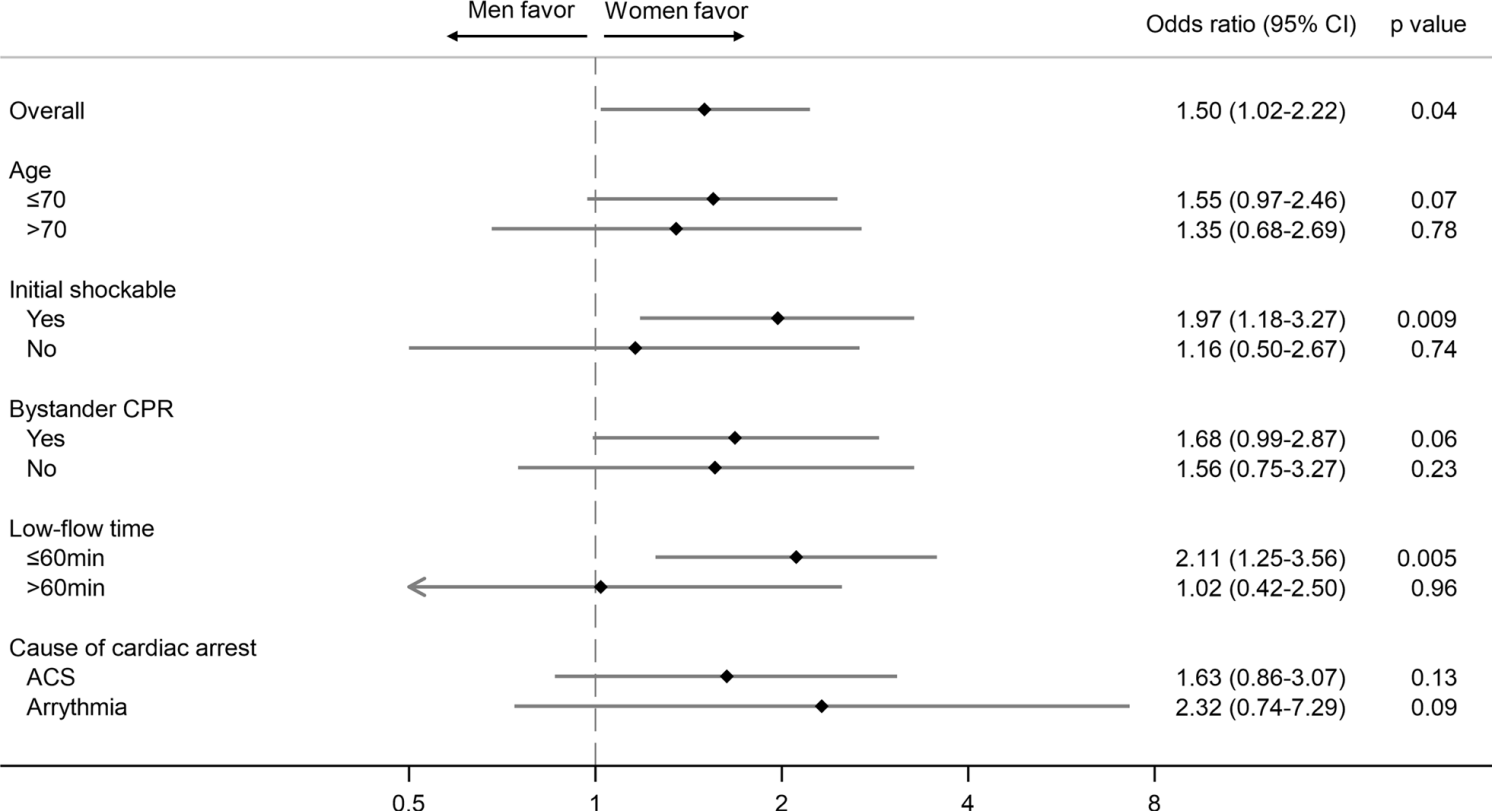
Subgroup analysis of primary outcome. CPR, cardiopulmonary resuscitation; ACS, acute coronary syndrome; CI, confidential interval

The proportion of favorable neurological outcomes was 12.3% in males and 15.9% in females (*p* = 0.10). The results of multilevel logistic regression analyses for primary outcome are shown in Fig. 2 and Tables S4 and S5. Women were significantly associated with a favorable neurologic outcome at discharge (adjusted OR: 1.60 [1.05–2.43], *p* = 0.03). The subgroup analysis showed a more apparent prognostic advantage for women in subgroups with younger age, initial shockable rhythm, bystander CPR, and shorter low-flow time (Fig. 3). Subgroup analysis based on the cause of cardiac arrest other than acute coronary syndrome and arrhythmia could not be performed due to the small sample size. Sensitivity analysis showed a tendency for women to have a prognostic advantage in each model.

### Other outcomes

The proportions of hospital survival (24.5% vs. 28.0%, *p* = 0.20) and decisions regarding WLST (27.1% vs. 29.7%, *p* = 0.36) were identical in both groups. The primary reason for WLST was an unfavorable prognosis, followed by the inability to sustain ECMO or manage complications. In the multilevel logistic regression analysis, the female sex was significantly associated with hospital survival (adjusted OR: 1.46 [1.03–2.06], *p* = 0.03). No significant differences were observed in severe AKI (adjusted OR: 0.67 [0.44–1.03], *p* = 0.07) or WLST (adjusted OR: 1.08 [0.78–1.50], *p* = 0.64).

## Discussion

### Key findings and strengths

Our study found that the female sex was significantly associated with favorable neurological outcomes at hospital discharge in Japanese patients with OHCA treated with ECPR. This prognostic advantage persisted across sensitivity and subgroup analyses.

The study’s strengths lie in providing robust evidence of sex-related differences in patients undergoing ECPR for OHCA. First, it focused exclusively on patients who underwent ECPR for OHCA, unlike previous studies that included heterogeneous patient populations. A large observational study using the ELSO registry, which included both OHCA and in-hospital cardiac arrest (IHCA), reported no sex differences in patient background or prognosis after ECPR [22]. However, this study lacked important pre-hospital variables that should be adjusted for in the analyses. Furthermore, the differences between OHCA and IHCA in ECPR, such as cardiac arrest situations, etiology, and outcomes, were substantial, making it unsuitable to apply the results from IHCA patients to OHCA patients [7, 26, 27]. Other observational studies from Taiwan and Germany that investigated sex differences in patients who underwent extracorporeal membrane oxygenation, included populations other than OHCA, such as those with IHCA or non-ECPR [28, 29]. Therefore, our research, which exclusively focused on patients with OHCA, provides novel insights into the literature on sex differences in patients requiring ECPR for OHCA.

Second, we employed several methodological approaches to ensure robust results in sex-related differences. We conducted several sensitivity analyses to select confounding variables rigorously, and to investigate the impact of ROSC on sex differences. Furthermore, we performed an additional analysis adjunctly adjusting for the occurrence of AKI, which differs between sexes and impacts neurologic outcomes [22–25]. In some sensitivity analyses, although the 95% confidence intervals only slightly overlapped with the null association, the point estimates consistently showed an advantage for women regarding prognosis. Notably, subgroup analysis indicated that prognostic advantage for women might be more apparent in candidates for ECPR with favorable factors, such as younger age, bystander CPR, initial shockable rhythm, or shorter low-flow time. Consequently, our results consistently demonstrate a robust prognostic advantage for women in neurological outcomes. Based on these findings, our study provides novel and detailed insights into sex differences in patient backgrounds and neurological outcomes among ECPR populations for OHCA.

### Clinical and research implication

There are two possible mechanisms to interpret these results. First, we considered that the potential disparity in patient selection for ECPR is a clinically essential mechanism. Gender treatment disparity is a crucial matter in OHCA situations. Women are less likely to receive bystander CPR, AED [30], and in-hospital interventions after ROSC, such as PCI and TTM, even when warranted [31]. Additionally, women’s lower socioeconomic status could decrease the chance of receiving required interventions [32], leading to sex differences in mortality in OHCA [33]. Therefore, a similar gender treatment disparity could exist in the implementation of ECPR. Women who undergo ECPR might represent a highly selected population with an expectation of favorable outcomes owing to specific social factors and decision-making processes. For example, women tend to communicate their advanced directives at the end of life more effectively to their families than men [34], and older women are less likely to prefer life-sustaining treatments [35]. Additionally, women often outlive their spouses, leading to more conservative treatment decisions by surrogate decision-makers who may not expect considerable recovery after extensive treatment [36]. These social factors and decision-making processes may result in women being more conservative in accepting aggressive interventions such as ECPR, opting for it selectively when a favorable neurological outcome is highly expected.

Another essential factor to consider is biological differences that might confer benefits to women among patients with OHCA treated with ECPR. Estrogen, a sex hormone responsible for the development and regulation of the female reproductive system, is expected to protect nerves owing to its anti-inflammatory and antioxidant effects, and stabilize mitochondrial membranes against ischemic brain injury [37–39]. Although the clinical benefits of these biological factors favoring women remain unclear, we speculate that these biological features may protect women from organ injuries and that men might tend to suffer from ischemic organ injuries more than women. For example, men experienced AKI after OHCA more frequently than women in a previous study and our cohort [22], supporting the hypothesis that women may exhibit greater resistance to ischemic organ injury, particularly in severely compromised patient groups. In addition, our subgroup analysis indicated that women in the younger group have slightly more prognostic advantage, which might support the relationship between fertility and organ organ-protective effect of female hormones.

Therefore, we consider that both gender treatment disparity in ECPR and biological advantages for women contribute to the implications of our results.

### Limitations

Our study had several limitations. First, although we addressed confounders in primary and sensitivity analyses, residual confounding by unmeasured factors, such as CPR quality or socio-economic status, may remain [32, 40]. These residual confounders might affect the results of our analyses. Second, we could not eliminate selection bias in the implementation of ECPR at each participating institution. Unfortunately, our registry lacked data on patients with OHCA who did not undergo ECPR. Thus, our study could not determine whether ECPR patient selection and treatment decisions were made without sex-related differences or gender disparity. Regarding sex disparities in patient selection in ECPR for OHCA, an observational study among Japanese patients with OHCA with an initial shockable rhythm reported no significant sex differences in ECPR implementation [41]. However, considering that women frequently present with an initial non-shockable rhythm in OHCA, which is sometimes ineligible for ECPR [42], further research on patient selection bias for ECPR is necessary to rigorously confirm our results and potential mechanisms. Third, the proportion of male patients was dominant in our registry, resulting in a limited number of female patients. Generally, males are dominant among OHCA patients, and our population may represent it. Although the estimated confidence interval was adequately narrow for interpretation, a larger number of female cases would provide a more accurate estimation. Fourth, our registry data was collected from participating facilities in Japan. Therefore, the race in the SAVE-J II registry was predominantly Japanese, and the emergency medical system was based on Japanese health care systems. Race may affect cardiac arrest outcomes [22, 43], and the applicability of ECPR largely depends on each institution’s resources [44]. A questionnaire survey for participating facilities in this registry revealed that the current ECPR practice, eligible patients, and protocols in Japan are slightly varied [45]. Therefore, our results may raise concerns about generalizability to other races and regions with different accessibility to ECPR, facility protocols, and healthcare systems. Finally, we had no access to laboratory data on sex hormones. Although the prognostic advantage in females might stem from hormonal mechanisms, we have no evidence to support this theory.

## Conclusion

The female sex is significantly associated with favorable neurological outcomes at hospital discharge in patients who received ECPR for OHCA. This prognostic advantage persisted across the subgroups and sensitivity analyses. Further research is warranted to ensure equitable ECPR implementation without sex disparity, thereby rigorously confirming the prognostic advantage for women receiving ECPR for OHCA.

## Supplementary Information

Additional file 1.

## Data Availability

Data used in this study are available from the SAVE-J II Study Group. However, data are available from the authors upon reasonable request and with the permission of the SAVE-J II Study Group.

## Abbreviations

AED: Automated external defibrillator
AKI: Acute kidney injury
BMI: Body mass index
CAG: Coronary angiography
ECMO: Extracorporeal membrane oxygenation
ECPR: Extracorporeal cardiopulmonary resuscitation
ICU: Intensive care unit
IHCA: In-hospital cardiac arrest
IABP: Intra-aortic balloon pumping
KDIGO: Kidney Disease Improving Global Outcomes
OHCA: Out-of-hospital cardiac arrest
PCI: Percutaneous coronary intervention
WLST: Withholding/withdrawal of life-sustaining therapy
