# Supplementary material for: Sex differences in extracorporeal cardiopulmonary resuscitation for out-of-hospital cardiac arrest: nationwide multicenter retrospective study in Japan
Source: Crit Care. 2024 Oct 31;28:302. doi: 10.1186/s13054-024-05086-9 (PMC11526675; doi:10.1186/s13054-024-05086-9)
Supplement: Supplementary file 1 — Additional file 1. [file 13054_2024_5086_MOESM1_ESM.docx]

Table S1: STROBE Statement for cohort studies

|  | **Item #** | **Recommendation** | **Page#** |
| --- | --- | --- | --- |
| **Title and abstract** | 1 | (*a*) Indicate the study’s design with a commonly used term in the title or the abstract | 1 |
|  |  | (*b*) Provide in the abstract an informative and balanced summary of what was done and what was found | 1-4 |
| **Introduction** | | |  |
| Background/rationale | 2 | Explain the scientific background and rationale for the investigation being reported | 4 |
| Objectives | 3 | State specific objectives, including any prespecified hypotheses | 6-7 |
| **Methods** | | |  |
| Study design | 4 | Present key elements of study design early in the paper | 7 |
| Setting | 5 | Describe the setting, locations, and relevant dates, including periods of recruitment, exposure, follow-up, and data collection | 8 |
| Participants | 6 | (*a*) Give the eligibility criteria, and the sources and methods of selection of participants. Describe methods of follow-up | 8 |
|  |  | (*b*) For matched studies, give matching criteria and number of exposed and unexposed | N/A |
| Variables | 7 | Clearly define all outcomes, exposures, predictors, potential confounders, and effect modifiers. Give diagnostic criteria, if applicable | 8 |
| Data sources/ measurement | 8* | For each variable of interest, give sources of data and details of methods of assessment (measurement). Describe comparability of assessment methods if there is more than one group | 9 |
| Bias | 9 | Describe any efforts to address potential sources of bias | 10 |
| Study size | 10 | Explain how the study size was arrived at | 10 |
| Quantitative variables | 11 | Explain how quantitative variables were handled in the analyses. If applicable, describe which groupings were chosen and why | 9 |
| Statistical methods | 12 | (*a*) Describe all statistical methods, including those used to control for confounding | 10 |
|  |  | (*b*) Describe any methods used to examine subgroups and interactions | 11 |
|  |  | (*c*) Explain how missing data were addressed | 10 |
|  |  | (*d*) If applicable, explain how loss to follow-up was addressed | N/A |
|  |  | (*e*) Describe any sensitivity analyses | 11-12 |
| **Results** | | |  |
| Participants | 13* | (a) Report numbers of individuals at each stage of study—eg numbers potentially eligible, examined for eligibility, confirmed eligible, included in the study, completing follow-up, and analysed | 12 |
|  |  | (b) Give reasons for non-participation at each stage | Fig.2 |
|  |  | (c) Consider use of a flow diagram | Fig.2 |
| Descriptive data | 14* | (a) Give characteristics of study participants (eg demographic, clinical, social) and information on exposures and potential confounders | 12-13 |
|  |  | (b) Indicate number of participants with missing data for each variable of interest | Table S2 |
|  |  | (c) Summarise follow-up time (eg, average and total amount) | 12-13 |
| Outcome data | 15* | Report numbers of outcome events or summary measures over time | 13-14 |
| Main results | 16 | (*a*) Give unadjusted estimates and, if applicable, confounder-adjusted estimates and their precision (eg, 95% confidence interval). Make clear which confounders were adjusted for and why they were included | Table S4  11 |
|  |  | (*b*) Report category boundaries when continuous variables were categorized | Table S2,3 |
|  |  | (*c*) If relevant, consider translating estimates of relative risk into absolute risk for a meaningful time period | N/A |
| Other analyses | 17 | Report other analyses done—eg analyses of subgroups and interactions, and sensitivity analyses | Fig2,3 |
| **Discussion** | | |  |
| Key results | 18 | Summarise key results with reference to study objectives | 14 |
| Limitations | 19 | Discuss limitations of the study, taking into account sources of potential bias or imprecision. Discuss both direction and magnitude of any potential bias | 19 |
| Interpretation | 20 | Give a cautious overall interpretation of results considering objectives, limitations, multiplicity of analyses, results from similar studies, and other relevant evidence | 16-19 |
| Generalisability | 21 | Discuss the generalisability (external validity) of the study results | 19 |
| **Other information** | | |  |
| Funding | 22 | Give the source of funding and the role of the funders for the present study and, if applicable, for the original study on which the present article is based | 23 |

Table S2: The number of observed and missing values

| Variable | Observed | Missing (%) |
| --- | --- | --- |
| Age | 1818 | 1 (0.06%) |
| Sex | 1819 | 0 |
| Body mass index | 902 | 917 (50.4%) |
| Past medical history | 1819 | 0 |
| Performance status | 1760 | 59 (3.2%) |
| Location of cardiac arrest | 1813 | 6 (0.3%) |
| Cause of cardiac arrest | 1818 | 1 (0.06%) |
| Witness | 1813 | 6 (0.3%) |
| Bystander CPR | 1791 | 28 (1.5%) |
| Automated external defibrillator | 1803 | 16 (0.9%) |
| Initial cardiac rhythm | 1802 | 17 (0.9%) |
| Low flow time | 1712 | 107 (5.9%) |
| Signs of life at hospital arrival | 1114 | 705 (38.8%) |
| pH at hospital arrival | 1720 | 99 (5.4%) |
| Percutaneous coronary intervention | 1762 | 57 (3.1%) |
| Intra aorta balloon pumping | 1819 | 0 |
| Targeted temperature | 1194 | 625 (34.3%) |
| Favorable neurologic outcome at discharge | 1819 | 0 |
| Hospital survival | 1819 | 0 |
| Acute kidney injury | 1414 | 405 (22.3%) |
| Withhold/withdraw life-sustaining therapy | 1819 | 0 |

Abbreviations: CPR, cardiac pulmonary resuscitation

Table S3: Comparison neurologic outcome at discharge and sex in patient backgrounds

|  | Male (n=1,523) | |  | Female (n=289) | |  |
| --- | --- | --- | --- | --- | --- | --- |
|  | Favorable neurologic outcome at discharge  (n=188) | No favorable neurologic outcome at discharge  (n= 1,335) | p-value | Favorable neurologic outcome at discharge  (n=47) | No favorable neurologic outcome at discharge  (n= 249) | p-value |
| Age (year) | 55.0 [44.5-66.0] | 61.0 [51.0-69.0] | <0.001 | 56.0 [46.0-69.0] | 60.0 [46.0-69.0] | 0.42 |
| Body mass index | 24.2 [21.6-26.3] | 24.7 [22.2-27.5] | 0.03 | 22.4 [19.9-24.8] | 23.9 [20.8-28.0] | 0.034 |
| Past medical history |  |  |  |  |  |  |
| Hypertension | 61 (32.4%) | 413 (30.9%) | 0.68 | 18 (38.3%) | 67 (26.9%) | 0.11 |
| Diabetes mellitus | 35 (18.6%) | 274 (20.5%) | 0.54 | 9 (19.1%) | 30 (12.0%) | 0.19 |
| Cardiac disease | 33 (17.6%) | 341 (25.5%) | 0.017 | 13 (27.7%) | 51 (20.5%) | 0.27 |
| Stroke | 8 ( 4.3%) | 85 ( 6.4%) | 0.26 | 3 ( 6.4%) | 16 ( 6.4%) | 0.99 |
| Chronic kidney disease | 8 ( 4.3%) | 72 ( 5.4%) | 0.51 | 4 ( 8.5%) | 6 ( 2.4%) | 0.034 |
| Performance Status* |  |  |  |  |  |  |
| 0 | 171 (91.0%) | 1,168 (87.5%) | 0.29 | 39 (83.0%) | 209 (83.9%) | 0.14 |
| 1 | 13 ( 6.9%) | 96 ( 7.2%) |  | 8 (17.0%) | 23 ( 9.2%) |  |
| 2 | 1 ( 0.5%) | 24 ( 1.8%) |  | 0 ( 0.0%) | 8 ( 3.2%) |  |
| Location of cardiac arrest |  |  |  |  |  |  |
| Home | 58 (30.9%) | 517 (38.7%) | 0.15 | 18 (38.3%) | 134 (53.8%) | 0.24 |
| Public place/Street | 65 (34.6%) | 436 (32.7%) |  | 11 (23.4%) | 49 (19.7%) |  |
| Workplace | 29 (15.4%) | 157 (11.8%) |  | 2 ( 4.3%) | 13 ( 5.2%) |  |
| Ambulance** | 26 (13.8%) | 132 ( 9.9%) |  | 13 (27.7%) | 39 (15.7%) |  |
| Other | 9 ( 4.8%) | 88 ( 6.6%) |  | 3 ( 6.4%) | 14 ( 5.6%) |  |
| Cause of cardiac arrest |  |  |  |  |  |  |
| Acute coronary syndrome | 121 (64.4%) | 748 (56.0%) | <0.001 | 19 (40.4%) | 82 (32.9%) | 0.1 |
| Arrhythmia | 29 (15.4%) | 162 (12.1%) |  | 10 (21.3%) | 32 (12.9%) |  |
| Myocarditis/Myopathy | 17 ( 9.0%) | 75 ( 5.6%) |  | 5 (10.6%) | 18 ( 7.2%) |  |
| Aortic dissection | 5 ( 2.7%) | 24 ( 1.8%) |  | 6 (12.8%) | 24 ( 9.6%) |  |
| Pulmonary embolism | 1 ( 0.5%) | 93 ( 7.0%) |  | 0 ( 0.0%) | 18 ( 7.2%) |  |
| Other diagnosed internal disease | 6 ( 3.2%) | 130 ( 9.7%) |  | 6 (12.8%) | 57 (22.9%) |  |
| Unknown etiology | 9 ( 4.8%) | 102 ( 7.6%) |  | 1 ( 2.1%) | 18 ( 7.2%) |  |
| Witness | 162 (87.1%) | 1,035 (77.6%) | 0.003 | 39 (84.8%) | 193 (78.1%) | 0.31 |
| Bystander CPR | 135 (73.0%) | 728 (55.3%) | <0.001 | 31 (67.4%) | 147 (60.5%) | 0.38 |
| Automated external defibrillator | 129 (69.4%) | 839 (63.2%) | 0.1 | 28 (62.2%) | 119 (48.6%) | 0.092 |
| Initial cardiac rhythm |  |  |  |  |  |  |
| Shockable rhythm | 157 (83.5%) | 871 (65.2%) | <0.001 | 34 (72.3%) | 110 (44.2%) | 0.003 |
| PEA | 25 (13.3%) | 334 (25.0%) |  | 11 (23.4%) | 104 (41.8%) |  |
| Asystole | 4 ( 2.1%) | 120 ( 9.0%) |  | 1 ( 2.1%) | 31 (12.4%) |  |
| Cardiac rhythm at hospital arrival |  |  |  |  |  |  |
| Shockable rhythm | 143 (76.1%) | 582 (43.6%) | <0.001 | 29 (61.7%) | 84 (33.7%) | 0.002 |
| PEA | 40 (21.3%) | 445 (33.3%) |  | 15 (31.9%) | 106 (42.6%) |  |
| Asystole | 5 ( 2.7%) | 304 (22.8%) |  | 3 ( 6.4%) | 58 (23.3%) |  |
| Cardiac rhythm at ECMO initiation |  |  |  |  |  |  |
| Shockable rhythm | 138 (73.4%) | 640 (47.9%) | <0.001 | 31 (66.0%) | 88 (35.3%) | 0.001 |
| PEA | 40 (21.3%) | 452 (33.9%) |  | 11 (23.4%) | 115 (46.2%) |  |
| Asystole | 8 ( 4.3%) | 233 (17.5%) |  | 5 (10.6%) | 38 (15.3%) |  |
| Time from emergency call to hospital arrival (min) | 29.0 [23.0-37.0] | 32.0 [26.0-39.0] | 0.002 | 33.5 [24.0-39.0] | 34.0 [28.0-39.0] | 0.23 |
| Low-flow time* (min) | 51.0 [41.0-62.0] | 55.0 [45.0-67.0] | <0.001 | 49.0 [41.0-57.0] | 56.0 [46.0-67.0] | 0.004 |
| Tips65 score** | 2.0 [2.0-3.0] | 2.0 [1.0-2.0] | <0.001 | 2.0 [1.0-3.0] | 1.0 [1.0-2.0] | 0.002 |
| Clinical characteristics at hospital arrival |  |  |  |  |  |  |
| Signs of life*** | 55 (51.4%) | 122 (14.6%) | <0.001 | 14 (51.9%) | 21 (14.6%) | <0.001 |
| Gasping | 50 (29.2%) | 92 ( 7.9%) | <0.001 | 10 (22.2%) | 18 ( 8.3%) | 0.006 |
| Pupillary light reaction | 22 (18.8%) | 68 ( 7.0%) | <0.001 | 6 (20.7%) | 14 ( 8.4%) | 0.045 |
| GCS M > 1 | 11 ( 5.9%) | 10 ( 0.8%) | <0.001 | 2 ( 4.3%) | 1 ( 0.4%) | 0.016 |
| Body temperature (℃) | 35.2 [34.4-35.8] | 35.2 [34.2-35.9] | 0.67 | 35.2 [34.1-35.8] | 35.4 [34.5-36.0] | 0.4 |
| pH | 7.0 [6.8-7.1] | 6.9 [6.8-7.0] | 0.006 | 7.0 [6.8-7.1] | 6.9 [6.8-7.0] | 0.16 |
| Lactate (mmol/L) | 13.4 [9.9-16.9] | 12.9 [10.1-16.0] | 0.43 | 12.5 [8.7-14.9] | 12.9 [10.2-15.0] | 0.31 |
| Coronary angiography | 175 (93.1%) | 971 (72.8%) | <0.001 | 42 (89.4%) | 158 (63.5%) | <0.001 |
| Percutaneous coronary intervention | 105 (59.0%) | 586 (45.3%) | <0.001 | 15 (32.6%) | 60 (24.7%) | 0.26 |
| Intra aortic balloon pumping | 145 (77.1%) | 791 (59.5%) | <0.001 | 30 (63.8%) | 117 (47.0%) | 0.034 |
| Target temperature (℃) |  |  |  |  |  |  |
| ≦34 | 115 (61.2%) | 547 (41.0%) | <0.001 | 26 (55.3%) | 93 (37.3%) | 0.002 |
| 35 | 19 (10.1%) | 72 ( 5.4%) |  | 5 (10.6%) | 16 ( 6.4%) |  |
| 36≦ | 31 (16.5%) | 226 (16.9%) |  | 10 (21.3%) | 34 (13.7%) |  |

Abbreviations: CPR, cardiac pulmonary resuscitation; ROSC, return of self-circulation; PEA, pulseless electrical activity; ECMO, extracorporeal membrane oxygenation; GCS, Glasgow Coma Scale-Motor

Table S4: Association of being female with outcomes in univariate model and multi-level logistic regression model in imputed data

|  | Univariate model | | Multi-level model | |
| --- | --- | --- | --- | --- |
|  | OR [95% CI] | p-value | OR [95% CI] | p-value |
| **Primary Outcome** |  |  |  |  |
| Favorable neurologic outcome at discharge |  |  |  |  |
| Primary analysis | 1.34 [0.95–1.90]– | 0.10 | 1.60 [1.05–2.43] | 0.03 |
| Sensitivity analysis |  |  |  |  |
| Pre-cardiac arrest factors model * |  |  | 1.44 [0.98–2.10] | 0.06 |
| Before ECPR factors model ** |  |  | 1.57 [1.03–2.38] | 0.04 |
| After ECPR run factors model*** |  |  | 1.50 [1.05–2.15] | 0.03 |
| ROSC patients included model**** | 1.24 [0.90–1.71] | 0.19 | 1.48 [1.00–2.19] | 0.049 |
| AKI adjusted model***** |  |  | 1.55 [1.02–2.37] | 0.04 |
| **Secondary Outcome** |  |  |  |  |
| Hospital survival | 1.20 [0.91–1.59] | 0.20 | 1.46 [1.03–2.06] | 0.03 |
| Severe acute kidney injury | 0.59 [0.39–0.89] | 0.01 | 0.67 [0.44–1.03] | 0.07 |
| Withhold/withdraw life-sustaining therapy | 1.14 [0.86–1.49] | 0.36 | 1.08 [0.78–1.50] | 0.64 |

OR: odds ratio; CI: confidence interval; ECPR: extracorporeal cardiopulmonary resuscitation; AKI: acute kidney injury

Multi-level logistic regression analysis adjusted with center level variable (hospital), and individual level variable (age, BMI, past medical history (hypertension, diabetes mellitus, cardiac disease, chronic kidney disease), performance status, location of cardiac arrest, witness, bystander CPR, initial cardiac rhythm, low-flow time, ROSC before cannulation, signs of life, cause of cardiac arrest, AED, pH at hospital arrival, PCI, IABP, and target temperature).

*: Age, BMI, past medical history, performance status, cause of cardiac arrest

**: Age, BMI, past medical history, performance status, cause of cardiac arrest, location, witness, bystander, AED, initial cardiac rhythm, low-flow time, ROSC before cannulation, signs of life, pH

***: PCI, IABP, target temperature

****: Model including patients with ROSC at hospital arrival and ECMO initiation

*****: AKI occurrence was added to the adjustment factors used in the primary analysis.

Table S5: Full model of multilevel analysis for favorable neurologic outcome at discharge with multiple imputation

|  | Odds ratio [95% CI] | p-value |
| --- | --- | --- |
| Age (year) | –0.03[–0.04–0.02] | <0.001 |
| Women | 1.60 [1.05–2.43] | 0.03 |
| Body mass index | –0.05 [–0.10–0.01] | 0.01 |
| Past medical history |  |  |
| Hypertension | 1.35 [0.95–1.93] | 0.09 |
| Diabetes mellitus | 0.98 [0.64–1.49] | 0.93 |
| Cardiac disease | 0.66 [0.44–0.99] | 0.04 |
| Chronic kidney disease | 1.31 [0.62–2.78] | 0.48 |
| Performance Status** |  |  |
| 0 | Reference |  |
| 1 | 1.22 [0.68–2.20] | 0.50 |
| 2 | 0.32 [0.04–2.54] | 0.28 |
| Location of cardiac arrest |  |  |
| Home | Reference |  |
| Public place/Street | 1.37 [0.93–2.02] | 0.11 |
| Workplace | 1.60 [0.95–2.69] | 0.08 |
| Ambulance | 1.66 [0.93–2.95] | 0.09 |
| Other | 0.81 [0.39–1.68] | 0.57 |
| Cause of cardiac arrest |  |  |
| Acute coronary syndrome | Reference |  |
| Arrhythmia | 0.93 [0.52–1.66] | 0.79 |
| Myocarditis/ Myopathy | 1.09 [0.54–2.19] | 0.80 |
| Pulmonary embolism | 2.10 [0.80–5.49] | 0.13 |
| Acute aortic dissection | 0.13 [0.02–0.98] | 0.05 |
| Other internal disease | 0.50 [0.23–1.08] | 0.08 |
| Unknown | 0.74 [0.33–1.67] | 0.47 |
| Witness | 1.56 [0.99–2.46] | 0.06 |
| Bystander CPR | 1.50 [1.05–2.14] | 0.03 |
| Initial cardiac rhythm |  |  |
| Shockable rhythm | Reference |  |
| PEA | 0.29 [0.17–0.51] | <0.001 |
| Asystole | 0.20 [0.07–0.52] | <0.001 |
| Low-flow time | –0.005 [–0.01–0.004] | 0.30 |
| Automated external defibrillator | 0.74 [0.49–1.13] | 0.16 |
| Signs of life at hospital arrival | 3.91 [2.70–5.66] | <0.001 |
| pH at hospital arrival | 1.24 [0.30–2.19] | 0.01 |
| Percutaneous coronary intervention | 1.05 [0.65–1.71] | 0.83 |
| Intra aorta balloon pumping | 1.55 [1.03–2.34] | 0.03 |
| Targeted temperature (℃) |  |  |
| <=34 | Reference |  |
| 35 | 1.27 [0.70–2.30] | 0.44 |
| 36<= | 0.82 [0.53–1.26] | 0.37 |

Abbreviations: CPR, cardiac pulmonary resuscitation; PEA, pulseless electrical activity
